# **The care episode of a young elite-level female cyclist with major depressive disorder and comorbid eating disorder: A sports psychiatry case report from Switzerland**

**DOI:** 10.1007/s44192-025-00242-1

**Published:** 2025-07-08

**Authors:** Michael Liebrenz, Christian Imboden, Jill Colangelo, Anna Buadze, Alexander Smith

**Affiliations:** 1 Department of Forensic Psychiatry, University of Bern, Hochulstrasse 4, Bern, 3012 Switzerland; 2 Private Clinic Wyss AG, Münchenbuchsee, Switzerland; 3 University Hospital of Psychiatry and Psychotherapy, University of Bern, Bern, Switzerland; 4 Psychiatric Services Solothurn, Solothurn, Switzerland; 5 Department of Psychiatry, Psychotherapy and Psychosomatics, Psychiatric Hospital, Specialized Clinic for ADHD, University of Zurich, Zurich, Switzerland

**Keywords:** Sports psychiatry, Athlete, Depression, Cycling, Eating disorder

## Abstract

Elite-level athletes can experience mental illnesses as frequently as the general population. In professional cycling, distinctive psychosocial risk factors and inadequate formal support may heighten psychiatric vulnerabilities, yet treatment case examples remain scarce. Consequently, we outline the care episode of a 20-year-old female elite-level cyclist in Switzerland, who ceased competing and was thereafter diagnosed with major depressive disorder and a continuing diagnosis for a comorbid eating disorder, likely exacerbated by sport-specific stressors. Whilst psychopharmacological outpatient treatment yielded initial progress, the patient’s persistent suicidality necessitated voluntary inpatient admission. Her mental health improved post-discharge, though reintegration into cycling entailed difficulties, prompting a transition to a team with a less demanding schedule that allowed for a better balance between sporting and personal commitments. At the time of writing, the patient’s symptoms had stabilised and she was engaging in advocacy to raise awareness about athlete mental health. Accordingly, this case adds to the therapeutic evidence-base on elite-level athletes, demonstrating the importance of individualised psychiatric interventions that prioritise sustainable and holistic wellbeing.

## Introduction

Despite often having superlative levels of physical fitness, elite-level athletes can exhibit psychiatric symptoms at rates comparable to the general population [1, 2]. Similar patterns have been identified in elite-level cycling, including a significant prevalence of depressive disorders, eating disorders (ED), substance use disorders, and other mental illnesses [3]. As a discipline, professional cycling can encompass complex psychosocial challenges, like competitive demands, weight-related concerns, injury fears, familial scrutiny (i.e., parents and other relatives pressurising the athlete to perform at a higher level), and regressive sociocultural attitudes towards mental health [3, 4]. These risk factors might be more pronounced in women’s elite-level cycling due to socioeconomic disparities and limited institutional support for riders, alongside other sport-specific phenomena [3, 5, 6].

Equally, socioenvironmental and practical barriers can impede access to psychiatric care amongst athletes [1, 2, 7]. Notably, in elite-level cycling, stigma, concerns about rider-team relationships, budget constraints, and prior anti-doping transgressions have potentially attenuated help-seeking behaviours [3–5]. Such considerations have likely contributed to the lack of clinical case descriptions about professional cyclists with mental illnesses, and, more broadly, about athletes with psychiatric disorders in different sports [3, 7]. Concomitantly, this has limited the development of evidence-based treatment schemes for athletes, as well as interventions that manage return-to-play decisions and promote sustainable wellbeing across the career span and beyond [3, 7, 8].

Accordingly, to help address this gap, we outline the case of a 20 year old elite-level female cyclist in Switzerland with major depressive disorder (MDD) and comorbid ED who required pharmacological interventions and inpatient care.

## Case description

### Psychosocial history

Born in 2004, the patient competed for a Union Cycliste Internationale (UCI) Continental Team (now classified as a UCI Women’s World Team), whilst excelling academically. She had a history of recurrent depressive symptoms but had no prior formal diagnosis for this disorder. However, she had been previously diagnosed with ED characterised by restrictive eating, which was treated with psychotherapy.

In the months before the care episode, her depressive symptoms had escalated in the context of familial criticisms and performance pressures and had impaired daily functioning. Specifically, she repeatedly interrupted training sessions to cry and contemplate suicide. Furthermore, her withdrawal from competitions due to mental ill-health provoked criticism from a family member that she had “already announced [her] resignation”, and she expressed disappointment over what she perceived to be was insufficient support and communication from the national cycling team.

### Clinical presentation, assessment, and diagnosis

The patient contacted the outpatient clinic for cyclists in at the Department of Forensic Psychiatry at the University of Bern in May 2024. Examined by the first author, she reported severe fatigue, impaired concentration, and suicidal ideation. Upon assessment, she scored 31 on the Beck Depression Inventory (BDI-II), thus indicative of severe depressive symptoms [9].

Additional cognitive and neuropsychological assessments were performed to evaluate the patient and determine the environments best suited for her future development, which in turn could inform return-to-play options and considerations about her overall wellbeing. This included the Wechsler Adult Intelligence Scale, the Auditory-Verbal Learning Test, the Rey-Osterrieth Complex Figure Test, the Tower of London Test, and the D2 Test of Attention [10–14]. The Minnesota Multiphasic Personality Inventory-2 Restructured Form (MMPI-2-RF), the Emotional Skills and Competence Questionnaire, and the Emotional and Social Competency Inventory were also administered to evaluate personality traits and the social competencies relevant to the patient’s athletic and academic pursuits [15–17].

Results from these assessments all fell within normative ranges. However, key insights derived from the automatic interpretation of the MMPI-2-RF indicated the presence of significant emotional distress, including cognitive disturbances and intrusive thoughts, which coincided with signs of interpersonal dysfunction, particularly in familial contexts.

In the initial examination, comprehensive laboratory tests were conducted and were repeated during the care episode to address potential somatic contributors and to track the patient’s physical health. Specifically, upon assessment, these excluded organic causes (e.g., hypothyroidism or anaemia). Furthermore, liver enzymes (e.g., aspartate aminotransferase, alanine aminotransferase, gamma-glutamyl transferase), kidney function markers, and serum albumin levels were all within normative parameters throughout the treatment course. In the first assessment, her Body Mass Index (BMI) was 22.0.

The patient was diagnosed with MDD (F33.1) and the continuation of an ED diagnosis of bulimia nervosa (F50.2) (non-purging subtype) based on the International Classification of Diseases categories [18]. Differential diagnoses of bipolar disorder and borderline personality disorder were considered because of dissociation and mood swings, but were ruled out due to insufficient clinical indicators.

### Treatment course

In May 2024, patient was prescribed with the selective serotonin reuptake inhibitor (SSRI), fluoxetine (20 mg daily), for its proven efficacy in treating MDD and reducing bingeing and other bulimia nervosa symptoms [19]. Early side-effects of insomnia and myoclonus were observed and were regularly monitored. The dosage was titrated to 40 mg per day according to clinical criteria. Nevertheless, the patient independently adjusted her medication dosage several times and informed the first author days after; specifically, she initially down-titrated in response to myoclonus and latterly up-titrated in an effort to accelerate psychiatric symptom amelioration. This required additional discussions about collaborative medication management, leading to agreed-upon morning and afternoon dosing schedules.

Despite early symptom improvements, the patient’s suicidality persisted, culminating in a crisis situation in June 2024 and a voluntary psychiatric inpatient admission; her inconsistent SSRI use patterns may have potentially contributed to this, together with continuing psychosocial stressors. In this setting, the patient received multimodal psychotherapy and continued with pharmacological treatment. Although in- and outdoor cycling training possibilities were available, she was encouraged to lessen her training load to reduce stress. In the care continuum, BDI scores were used to chart symptomatological trajectories, supplemented by the Symptom Checklist-90 [20]. Post-discharge, by mid-July 2024, her depressive symptoms had substantially decreased (BDI-II: 13).

### Outcomes and follow-up

At the time of submission, the patient was treated with fluoxetine at 60 mg daily, exhibiting good tolerance, and her MDD and ED symptoms had stabilised. Throughout the described care episode, her BMI had not changed significantly, which may have been influenced by the physiological demands of cycling. She continues regular consultations with the first author and a sports psychologist.

That said, initially, ongoing psychosocial stressors following inpatient treatment provoked challenges for her reintegration into the sport. By September 2024, she had joined a continental-level team with a more proactive approach towards mental health and a less demanding schedule, enabling her to better balance athletic and academic commitments. This team agreed to a contract permitting race participation contingent on the patient’s condition, and she has also publicly discussed her experiences to help destigmatise mental health in sports. Subsequently, in November 2024, she recorded BDI-II scores of 14 and 13, signifying mild-to-no depression based on this scale.

## Discussion

Although centred on a single episode of care, this case contributes to the evidence-base in sports medicine and sports psychiatry, where therapeutic reports focussing on athlete mental health remain scarce [1, 3, 7]. The necessity of inpatient treatment in this case underlines the potential implications of psychiatric symptoms amongst elite-level athletes, reinforcing how these individuals can be equally susceptible to severe mental health conditions as the general population [1]. Consequently, akin to different athletes, the patient’s advocacy in the media could help shape societal attitudes in Switzerland and elsewhere, bringing greater attention to the mental health challenges in professional sports [21].

From a clinical perspective, this case also illustrates the potential difficulties in treating athlete-patients with distinctive physiology. For example, as a weight-sensitive sport, lower BMIs often confer ergometric advantages in professional cycling due to its endurance demands and emphasis on altitude gain, and may therefore be incentivised amidst competitive environments. Whilst this patient’s BMI consistently fell within a normative range throughout the care episode, lower BMIs have been found to have associations with depression, notably in female samples [22, 23].

Moreover, myoclonus in the form of sudden muscle jerks can be a common SSRI side-effect, as can extrapyramidal reactions, which may manifest in thirty days of treatment initiation [24, 25]. Given metabolic factors, the patient’s body composition as an elite-level cyclist could have conceivably influenced drug pharmacokinetics (e.g., serum concentrations and clearance) and pharmacodynamics due to musculoskeletal differences and increased training load compared to non-athlete patients. This serotonergic activity variation might have increased her vulnerability to these issues, and led to her down-titrating the SSRI dosage, which in turn could conceivably have contributed to her requiring inpatient care.

Additionally, the patient’s pre-treatment experiences appear reflective of common socioenvironmental risk factors reported in elite-level cycling. This included her exposure to performance-based and familial pressures, which have been identified as psychosocial stressors for female riders [3, 5, 6]. As this case exemplifies, late psychiatric referral and impediments for help-seeking can further exacerbate these problems but are still recurring phenomena in sporting contexts [1].

Routine mental health screening and psychoeducation schemes may mitigate these barriers, but their success will be contingent on commitments from cycling teams, sports physicians, and other stakeholders [3, 5]. Correspondingly, achieving these systemic changes may entail difficulties in elite-level cycling due to stigma and regressive sociocultural attitudes about pain and endurance [3–6]. In this regard, the patient highlighted a lack of team and institutional support around her mental health, as has previously been illustrated in women’s professional cycling [5].

More broadly, this case reemphasises the importance of considering flexible, non-clinical outcomes in sports psychiatry. Specifically, it underlines how psychiatric-specific return-to-play considerations should be individualised as recovery is often nonlinear [8]; simply put, for some athletes, rigid and high-pressure environments may undermine their overall quality of life. For this patient, transitioning to a new team enabled her to find a healthier balance between athletic and personal priorities, as demonstrated by the later BDI-II scores in November 2024, thus accentuating one goal of sports psychiatry in promoting sustainable wellbeing over short-term performance.

## Conclusion

This case illustrates how severe psychiatric disorders can manifest in sporting settings, particularly in women’s elite-level cycling where there may be complex psychosocial risk factors and distinctive physiological demands. Further, this care episode foregrounds the possible implications of mental illnesses in athlete populations, reinforcing the necessity of timely and personalised interventions to promote sustainable wellbeing, especially within the context of return-to-play deliberations.

Ultimately, mandating regular mental health screening and strengthening stigma reduction programmes in women’s cycling would be important facilitators for broader access to tailored care; to that end, it is hoped that this patient’s openness can contribute to necessary shifts within women’s cycling and wider sporting environments.

## Data Availability

This manuscript does not report data generation or analysis.
